# An Overview of Long Noncoding RNAs Involved in Bone Regeneration from Mesenchymal Stem Cells

**DOI:** 10.1155/2018/8273648

**Published:** 2018-01-28

**Authors:** Shuping Peng, Lihua Cao, Shiwei He, Yancheng Zhong, Haotian Ma, Yanru Zhang, Cijun Shuai

**Affiliations:** ^1^Hunan Provincial Tumor Hospital and The Affiliated Tumor Hospital of Xiangya School of Medicine, Basic Medicine College, Central South University, Changsha 410013, China; ^2^Cancer Research Institute, School of Basic Medical Science, Central South University, Changsha 410078, China; ^3^School of Energy and Mechanical Engineering, Jiangxi University of Science and Technology, Nanchang, Jiangxi, China; ^4^State Key Laboratory of High Performance Complex Manufacturing, College of Mechanical and Electrical Engineering, Central South University, Changsha, Hunan, China

## Abstract

Bone regeneration is very important for the recovery of some diseases including osteoporosis and bone fracture trauma. It is a multiple-step- and multiple-gene-involved complex process, including the matrix secretion and calcium mineralization by osteoblasts differentiated from mesenchymal stem cells (MSCs) and the absorption of calcium and phosphorus by osteoclasts differentiated from hematopoietic stem cells. Long noncoding RNAs (lncRNAs) are a family of transcripts longer than 200 nt without or with very low protein-coding potential. Recent studies have demonstrated that lncRNAs are widely involved in the regulation of lineage commitment and differentiation of stem cells through multiple mechanisms. In this review, we will summarize the roles and molecular mechanism of lncRNAs including *H19*, *MALAT1*, *MODR*, *HOTAIR*, *DANCR*, *MEG3*, *HoxA-AS3*, and *MIAT* in osteogenesis ossification; lncRNA *ZBED3-AS1* and *CTA-941F9.9*, *DANCR*, and *HIT* in chondrogenic differentiation; and lncRNA *DANCR* in osteoclast differentiation. These findings will facilitate the development and application of novel molecular drugs which regulate the balance of bone formation and absorption.

## 1. Introduction

The bone regeneration after bone fracture trauma or other diseases is a process participated by a well-organized system of the synergistic effect of MSCs, immune cells, and osteoclasts. Osteoclasts absorb the organic and inorganic compounds released from the impaired bone, during which the degraded compound matrix goes into the bloodstream in the form of Ca2^+^, (PO4)3^−^, and so on for recycling [1, 2]. Meanwhile, the cytokines after the damage process initiate the osteogenic differentiation of MSCs. MSCs gradually differentiate into osteoprogenitors, preosteoblasts, and osteoblasts. The well-differentiated osteoblasts synthesize and secrete the matrix and thus induce the initiation of bone formation. MSC-mediated bone regeneration and osteoclast-mediated bone resorption are the two core processes of bone regeneration and repair. The process of osteogenic differentiation of MSCs is mainly regulated by tissue-specific transcriptional regulators and epigenetic factors [3, 4]. On the one hand, in the corporate induction of BMPs, Wnt/*β*-FGF, and other growth factors, related molecules in the signal pathways such as BMPs/Smads [5], Wnt/*β*-catenin [6] and MAPK/p38 [7], and transcription factors RUNX2 [8] and OSX [9] are activated, increasing the expression of osteoblast-specific genes (*OPN*, *OCN*, *ALP*, and *COL1A1*); eventually, MSCs differentiate into osteoblasts. On the other hand, epigenetic modulation including DNA methylation, histone modification, and noncoding RNA regulation also exerts a role in the regulation of osteogenic differentiation of MSCs. The regulation of DNA methylation and histone modification has been well understood. For example, Dansranjavin et al. found that the osteocalcin of undifferentiated stem cells was hypermethylated. However, in mature osteocytes, the degree of methylation was reduced and the expression levels of osteocalcin were increased [10]. Hsiao et al. observed that transfection of human bone marrow MSCs with the methylated thyroid hormone receptor interactor 10 (Trip 10) promoter resulted in cytosine methylation at the promoter region and downregulation of Trip10 expression, in which accelerating MSCs differentiate into neurons and osteoblasts [11]. Histone acetylation and methylation are another important epigenetic mechanisms in the process of osteogenesis [12–15]. Studies have shown that the BMP signaling pathway promotes osteogenic differentiation by regulating the acetylation of H3K9. Shen et al. observed that H3K4 methylation decreased while H3K9 acetylation increased during the osteogenic differentiation of ROS17/2.8 osteosarcoma cells and normal osteoblasts by using CHIP-seq techniques [16]. The roles and related mechanisms of miRNAs in bone development and balance have been reviewed [17–19]. However, the regulation of lncRNAs on bone regeneration has not well summarized.

Long noncoding RNAs (lncRNAs) belong to a family of transcripts longer than 200 nt without or with very low protein-coding potential. In the human genome, 15,787 lncRNA transcripts from 14,470 lncRNA genes have been identified, while the GENCODE annotation is constantly being updated [20, 21]. It is believed that lncRNAs are a transcriptional noise for a long time, which are a byproduct of RNA polymerase II transcription without biological function. However, recent studies have found that lncRNAs play a crucial role in regulating nuclear chromatin structure and gene expression in the developmental process and are also an active participant in disease occurrence and development [22–24]. Except extensive and constitutive expression of partial lncRNAs, most lncRNAs are specifically expressed during the cell tissue developmental stage. In general, the general expression levels of lncRNA are lower than those of mRNA. Some lncRNAs are located in the cell nucleus and some in the cytoplasm. Compared with miRNA, the interspecies homology similarity of lncRNAs is relatively lower, but there is a certain degree of conservation in its promoter region and exon area, which indicates the function of lncRNAs is relatively conservative. The transcripts produced from the 4~9% sequence of the mammalian genome sequence are lncRNAs (corresponding protein-coding portion is 1%). Despite that the recent advances on lncRNA have progressed rapidly, the functions of most of lncRNAs are still unclear.

## 2. Classification and Characteristics of lncRNAs

According to the genomic location, lncRNAs can be classified into five types: sense, antisense, bidirectional, intronic, and intergenic [3, 4, 25]. Many lncRNAs have conserved secondary structures, alternative splicing, and subcellular localization. The conservativeness and specificity indicate that they are functional [26]. lncRNAs possess the following characteristics: (1) The length of transcripts is 200–100,000 nt, with a similar structure to that of the mRNA. After splicing, there is a structure with a poly(A) tail and a promoter. During differentiated processes, there are a dynamic expression mechanism and alternative splicing that form different lncRNAs [27]. (2) Generally, lncRNAs have noncoding potentials, but some lncRNAs can encode some short peptides [28]. (3) They have low conservation [29]. (4) They are tissue-specific and spatiotemporal-specific. The amount of lncRNAs expressed in different tissues was different, and the expression of lncRNAs was different in the same tissues but different status [30]. (5) The abundance of different lncRNAs is various in different cells [31].

## 3. Modes of Action of lncRNAs

With the gradual knowledge of lncRNA functions, the mechanism of lncRNA interaction with targets has become a hot topic. Early identification of in situ regulation is the only mechanism in which lncRNAs silence the transcription of adjacent genes by recruiting chromatin-modifying complexes. The mechanism of lncRNAs is very complex and has not yet been fully understood. According to the current research, the mechanism of lncRNAs could be summarized as the four levels (epigenetic, transcriptional, and posttranscriptional regulation and other specific regulation modes).

### 3.1. lncRNAs Mediate Epigenetic Modifications

lncRNAs can recruit a chromatin remodeling complex to specific sites and then regulate the expression of targeting genes. For example, HOTAIR derived from the HOXC loci recruits the chromatin remodeling complex PRC2 and locates it to the HOXD site, thereby inducing the parent genetic silencing of the HOXD loci [32–34]. Similarly, lncRNAs *Xist* [35] and *Kcnq1ot* [36, 37] can be recruited by the remodeling complexes such as methyltransferase Ezh2 or G9a to realize epigenetic silence of related genes.

### 3.2. lncRNAs Regulate Transcriptional Expression

lncRNAs can silence gene expression at the transcriptional level through a variety of mechanisms. lncRNAs can interfere with the transcription of adjacent genes. For example, in yeast, the transcription of the *SER3* gene is affected by its upstream lncRNA *SRG1* [38]. lncRNAs can interfere with gene expression by blocking the promoter region. For instance, lncRNA *DHFR* can form an RNA-DNA3 helix structure in the promoter region of the *DHFR* gene [39], inhibiting the binding of the transcription factor TFIID and thereby inhibiting *DHFR* gene expression. Moreover, lncRNA can interact with RNA-binding proteins and target to the promoter region, regulating gene expression. For instance, lncRNA located in the upstream of the *CCND1* promoter can regulate the activity of the RNA-binding protein TLS and affect the expression of *CCND1* [40]. Besides, lncRNAs regulate the activity of transcription factors. lncRNA *Evf2* can form transcriptional complexes with the transcription factor Dlx2 to activate Dlx6 expression [41, 42]. At last, lncRNAs can control gene expression by regulating the basic transcription factor. For instance, *Alu* RNA can realize extensive gene suppression by inhibiting RNA polymerase II [43].

### 3.3. lncRNAs Mediate Posttranscriptional Regulation

lncRNA can form double-stranded RNA complexes with mRNA at the posttranscriptional level to mask the major *cis*-acting elements of mRNA, thereby regulating gene expression. For example, lncRNA *Zeb2* (Sip1) is able to form a double strand at the 5′ end shear site of an intron of the mRNA transcribed by the HOX site, thereby preventing the intron from being sheared. The region contains ribosome-binding sites which are necessary for the expression of Zeb2 protein, and Zeb2 antisense RNA can increase the expression of Zeb2 protein in this way. This example shows that lncRNAs can guide alternative splicing of mRNA isoforms. lncRNAs compete with mRNA to bind miRNA-binding sites, leading to the upregulation of miRNA target molecules. Lnc*MD* as a spongy molecule isolates *miR-125b* binding to the target molecule *IGF* mRNA, promoting MSC differentiation into muscle cells [44].

### 3.4. Other Specific Regulation Modes

In addition, the renaturation (annealing) of lncRNAs has a targeting effect, allowing protein receptor complexes to recognize the mRNA transcripts of the sense chain. This mode resembles the RNA-induced silencing complex (RISC) targeting mRNA through siRNA. Double-stranded RNA derived from complementary transcripts and even lncRNA, combined with extended internal hairpin structure, can be processed into endogenous siRNA to silence gene expression.

## 4. lncRNAs in Bone Development and Homeostasis

The formation of new bone is induced from MSCs via lineage commitment, which successively form osteoprogenitor cells, preosteoblasts, mature osteoblasts, and osteocytes. These major regulatory mechanisms, including tissue-specific transcription factors and regulatory molecules, mediated bone matrix synthesis, bone remodeling, and mineralization-related and repair-related gene expression. These osteogenic activities are simultaneously regulated by genetic and epigenetic levels. Epigenetic regulation includes DNA methylation, histone modification, and miRNA and lncRNA regulation. miRNA regulation for bone function and repair process has been summarized before.

Numerous experiments have shown that lncRNAs play a role in these processes. Classification and functional analyses also show that lncRNAs are involved in the lineage differentiation of MSCs into muscle cells, adipocytes, chondrocytes, and osteoblasts. Many scientists have focused on how lncRNAs play a role in stem cell differentiation for the past few years. Here, we will concentrate on the expression of lncRNAs in osteogenic differentiation of MSCs (Table 1). In addition, we will also analyze the role of lncRNAs in bone and cartilage differentiation, as well as the role and balance of bone, cartilage, and osteoclasts.

## 5. Global Transcriptomic Analyses Identify lncRNA Profiles

Zuo et al. [45] firstly published the earliest report about osteogenesis-related lncRNAs identified at 2013. They found that the expression profile of lncRNAs from C3H10T1/2 MSCs was changed under BMP2 induction. At the same time, they identified 116 differentially expressed lncRNAs and these lncRNAs positively regulate the expression of its adjacent genes, which indicated that lncRNAs regulated osteogenesis under the synergistic effect of adjacent genes. Song et al. utilized high-throughput RNA sequencing (RNA-seq) data to detect the expression profile of lncRNAs from immortalized MSCs which was induced by osteogenic induction medium for 28 days and thus screened 2597 mRNAs and 574 lncRNAs, of which 351 were known lncRNAs and 217 were novel lncRNAs. 32 novel lncRNAs are the precursor molecules of *miR-689*, *miR-640*, *miR-601*, and *miR-544*. They also constructed 14,275 coexpression relationships in the osteogenesis process, as well as 217 gene regulatory networks between the novel lncRNA and the mRNA [46] .

Qu et al. utilized high-throughput expression profiles (30,586 lncRNAs and 26,109 coding transcripts) to screen the differentially expressed genes of human periodontal ligament stem cells (hPDLSCs), which were, respectively, cultured in growth medium and osteogenic induction medium for 14 days, and screened out 3557 differentially expressed mRNAs, among which 1578 mRNAs were upregulated, 1979 mRNAs downregulated, 994 lncRNAs upregulated, and 1177 lncRNAs downregulated. These lncRNAs (*AC078851.1*, *RP11-45A16.4*, *XLOC_002932*, *RP4-613B23.1*, and *RP11-305L7.6*) and mRNAs (*ALP*, *COL1A1*, and *COL1A20*) are upregulated and BMP5 and IL6 are downregulated as verified by Q-PCR indicating that there are 131 pairs of lncRNA-mRNA regulatory relationships and 262 pairs of positive regulatory relationships, and MAPK, VEGF, and TGF-*β* signaling pathways were mainly involved in the regulation during osteogenic differentiation process [47].

Zhang et al. reported the human BMSCs derived from 18- to 20-year-old healthy male bone marrow cultured in osteogenic induction medium for 7 days and screened them with high-throughput human transcription microarray (Affymetrix, covering more than 285,000 coding and 40,000 noncoding transcripts). They screened out 1269 differentially expressed mRNAs (among which 648 were upregulated and 621 were downregulated) and 1408 lncRNAs, and MAPK, JAK-STAT, Toll-like receptor, and TGF-*β* signal pathways were found to participate in osteogenic differentiation of hBMSCs. GPX3, TLR2, BDKRB1, FBXO5, BRCA1, MAP3K8, SCARB1, and 6 lncRNAs (*XR_111050*, *NR_024031*, *FR374455*, *FR401275*, *FR406817*, and *FR148647*) played a key role in osteogenic process, and lncRNA *XR_111050* promoted osteogenic differentiation of mesenchymal stromal cells [48]. It can be confirmed that lncRNAs play an important role in osteogenesis differentiation, and the current studies have identified a number of differentially expressed lncRNAs. However, the mechanisms of most lncRNAs regulating the osteogenesis process remain to be understood and explored.

## 6. lncRNAs Are Functionally Involved in Bone Development and Homeostasis

### 6.1. lncRNAs That Promote Osteogenic Differentiation of Stem Cells

#### 6.1.1. H19

**Figure 1:**
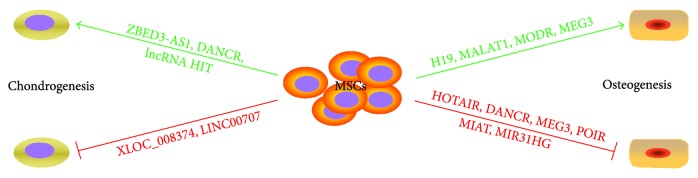
lncRNAs that promote osteogenic differentiation of stem cells.

*H19* (imprinted maternally expressed transcript) is one of the highly upregulated genes during the induction of primitive stem cells with osteogenic induction medium. It is located on 11p15.5 and is 2.3 kb in length and is conserved in evolution and plays an important role in regulating biological functions. *H19* is the precursor of *miR-675* which can generate two mature miRNAs (*miR-675-5p* and *miR-675-3p*) by Drosha and Dicer with splicing-dependent modes. *H19* and *miR-675* were upregulated during osteogenic differentiation of human MSCs. *miR-675* not only downregulates TGF-*β*1 but also inhibits Smad3 phosphorylation and downregulates HDAC4/5 leading to reduced HDACs to be recruited to the promoter of osteogenesis-specific runt-related transcription factor 2 (Runx2) [49]. Liang et al. found that *H19*, acting as an endogenous competitive ceRNA for *miR-141* and *miR-22*, directly binds to *miR-141* and *miR-22* preventing the inhibition of *miR-141* and *miR-22* on Wnt/beta-catenin, thereby promoting osteogenic differentiation [50]. Huang et al. reported that *H19* and *miR-675* overexpression inhibited adipogenic differentiation. *miR-675* targets to the 3′-UTR region of histone deacetylase HDAC4~6 that downregulates the expression of HDAC4~6 which is the essential molecules of fat differentiation. Recent studies have shown that *H19* plays a critical role in embryonic placenta growth and cell differentiation [51] (Figure 1).

#### 6.1.2. MALAT1

*MALAT1* (metastasis-associated lung adenocarcinoma transcript 1) is firstly found to be positively correlated with metastasis in lung adenocarcinoma, which is located on 11q13.1 and is 8545 nt in length. Xiao et al. demonstrated that *MALAT1* promotes osteogenic differentiation of aortic valve interstitial cells in calcific aortic valve disease (CAVD). Further studies showed that *MALAT1* functioned as a sponge molecule of *miR-204* and upregulated the expression of Smad4. Smad4 activation promotes the expression of alkaline phosphatase and downstream molecule osteocalcin and thus promotes bone formation and mineralization [52] (Figure 1).

#### 6.1.3. MODR

lncRNA *MODR* is an upregulated lncRNA in the process of osteogenesis of maxillary sinus membrane stem cells. Silencing lncRNA *MODR* can reduce the expression of *RUNX2*. lncRNA *MODR* acts as a molecular sponge for binding to *miR-454* to relieve its inhibition for *RUNX2*, thus upregulating *RUNX2* expression and promoting osteogenesis [53] (Figure 1).

### 6.2. lncRNAs That Inhibit Osteogenic Differentiation

#### 6.2.1. HOTAIR

**Figure 2:**
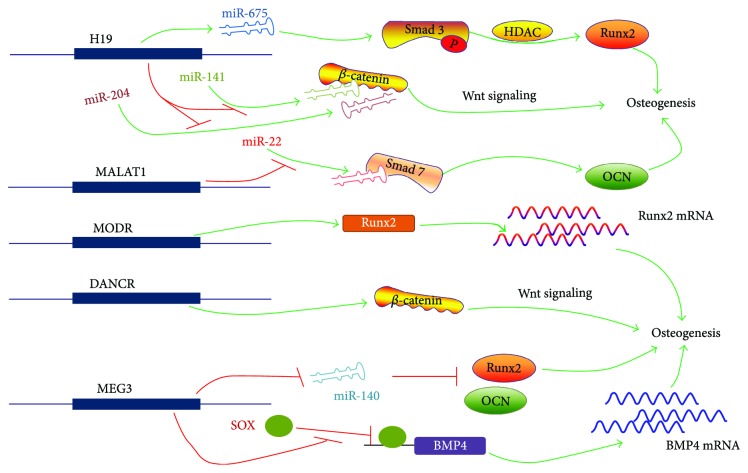
lncRNAs that inhibit osteogenic differentiation.

*HOTAIR* (HOX transcript antisense RNA), which is 2.2 kb in length and is located on 12q13.13, is a long noncoding RNA formed by *HOXC* gene transcription. Gene knockout of *HOTAIR* can lead to homologous transformation and skeletal malformations. *HOTAIR* can inhibit *HOXD*, *HOTAIR*, and imprinted loci, such as DLK1-MEG3 and Igf2-H19. At the same time, it combines with polycomb repressive complex 2 (PRC2, methylated H3k27) and Lsd1 complex (LSD1, demethylated H3K4), resulting in an increase in H3K27me3 expression and a decrease in H3K4me3 expression, indicating that HOTAIR can enhance the inhibition of HOX and other target genes by chromatin remodeling. Homozygous deletion of the *HOTAIR* gene in mice leads to lumbosacral bone turnover and metacarpal and carpal bone fusion, which is similar to the ectopic overexpression of *HOXD* in transgenic mice with an increase in *HOXD10* and *HOXD11* expression, suggesting that *HOTAIR* transregulated *HOXD* gene expression by recruiting polycomb and inhibiting the PRC2 complex targeting *HOXD* sites [32]. Compared with osteoarthritis, the expression of the long noncoding RNA *HOTAIR* from MSCs of patients with nontraumatic necrosis of the femoral head is upregulated while that of *miR-17-5p* was downregulated. Knockdown of *HOTAIR* by siRNA leads to an increase in *miR-17-5p* expression and a decrease in its target gene smad7, while *RUNX2*, COLA1 mRNA, and alkaline phosphatase activities were upregulated. Thus, *HOTAIR* reduces the expression of *miR-17-5p* by reducing Smad7 and then inhibiting osteogenic differentiation [54] (Figure 2).

#### 6.2.2. DANCR

*DANCR* (differentiation antagonizing non-protein-coding RNA) was initially found to be downregulated during calcium ion-induced primary human keratinocyte differentiation by RNA-seq. Human *DANCR* is located on 4q12 and is 915 bp in length, having 3 exons and an miR-4449-binding site, and located at the downstream of 54.8 kb and 28.7 kb are the *USP* and *ERVMER34–1* genes. Zhu and Xu later found that downregulation of *DANCR* promoted osteogenic differentiation of the human fetal osteoblastic cell line hFOB1.19 cultured in osteogenic induction medium. They further found that a transcript with a length of 305 nt at the *DANCR* 3′ end interacted with *EZH2* (enhancer of zeste homolog 2), whereas *DANCR* recruited *EZH2* to promote H3K27me3, which inhibited target gene *RUNX2* transcription and osteogenic differentiation [55]. Jia et al. studied that downregulation of *DANCR* in periodontal ligament stem cells promotes osteogenic differentiation by activating classical Wnt signaling pathways [56] (Figure 2).

#### 6.2.3. MEG3

Zhuang et al. found that *MEG3* (maternally expressed 3) promotes the differentiation of bone marrow stem cells (hBMSCs) into osteoblasts in patients with multiple myeloma. *MEG*3, which is located on 14q32.2 and is 1595 bp in length, promotes the translation of BMP4 located at the downstream apart few Mbs by preventing the inhibiting effect of *SOX2* in the BMP4 promoter region [57]. However, Li et al. detected that MEG3 expression is downregulated during adipose-derived MSC differentiation into adipogenic cells and upregulated during osteogenic differentiation. Knockdown of MEG3 promotes the osteogenic and adipogenic differentiation of human adipose-derived MSCs [58]. The mechanism may be associated with *miR-140-5p*. It also has been reported that *MEG3* upregulated *miR-133a-3p* and inhibited osteogenic differentiation in bone marrow MSCs from patients with postmenopausal osteoporosis [59] (Figure 1).

#### 6.2.4. HoxA-AS3

*HoxA-AS3* (HOXA cluster antisense RNA 3) was originally identified as an upregulated molecule in glioma patients, which is located on 7p15.2 and is 3992 nt in length. Later, *HoxA-AS3* was found to inhibit osteogenic differentiation of human bone marrow-derived stem cells (hBMSCs) and promote adipogenic differentiation [60]. *HoxA-AS3* inhibits *RUNX2* transcription by binding to *EZH2* and promoting H3k27 methylation. *HoxA-AS3* acts as an epigenetic modified switch to inhibit osteogenic differentiation of MSCs [61] (Figure 2).

#### 6.2.5. MIAT

*MIAT* (myocardial infarction-associated transcript) is located on chromosome 22q12.1 and downregulated in human adipose-derived stem cells (hASCs) during osteogenic differentiation. Knockdown of *MIAT* promoted osteogenic differentiation of hASCs *in vitro* and *in vivo*. Tumor necrosis factor treatment increases *MIAT* expression. Knockdown of *MIAT* can reverse the inhibition of osteogenic differentiation induction by an inflammatory factor. It acts as a sponge molecule of *miR-150-5p* to regulate its binding to the target gene and also acts as an endogenous competitive RNA to form AKT-miR-150-5p feedback loop to regulate oxidative stress and inflammatory factors and to stimulate the functional regulation of human lens epithelial cells [62–64] (Figure 2).

#### 6.2.6. POIR

lncRNA *POIR*, which is located on chromosome 6 and is 786 nt in length, was found to be expressed differentially in periodontal MSCs from patients with periodontitis and healthy human. Its expression is upregulated during osteogenic differentiation of periodontal membrane stem cells (PMSCs). Further studies have shown that lncRNA *POIR* as an endogenous competitive RNA competes for the binding sites of *miR-182*, leading to an increase in its target gene *FOXO1*. *FOXO1* promotes bone formation by inhibiting the classical Wnt signaling pathway by competing with TCF-4 for beta-catenin. And abnormal activation of the NF-*κ*B pathway during inflammation can increase the level of *miR-182* and decrease the level of lncRNA *POIR*, which breaks the balance of lncRNA-POIR-miR-182 regulatory network [65] (Figure 1).

#### 6.2.7. MIR31HG

*MIR31HG* (MIR31 host gene) is located on 9p21 and is 745 nt in length, which is found downregulated by Jin et al. in the process of induced human adipose-derived stem cell (hASC) osteogenic differentiation. Knockdown of *MIR31HG* not only promotes the formation of bone but also overcomes the inflammatory inhibition of the osteogenesis process. From the mechanism, it was found that *MIR31HG* interacted with NF-*κ*B where p65 subunit bound to the *MIR31HG* promoter region and promoted *MIR31HG* expression. *MIR31HG* binds directly to I*κ*B*α* and participates in NF-*κ*B activation. Thus, targeting to the MIR31HG-NF-*κ*B regulatory loop can improve the osteogenic ability of hASCs in inflammatory environments [66] (Figure 2).

## 7. lncRNAs Involved in Chondrogenic Differentiation

Cartilage plays a role in modeling, protecting and supplementing bone tissue during individual development. Chondrocytes are derived from bone pluripotent precursor cells and can form the primordial cartilage and are the main skeleton of the embryo by directional specific regulatory systems and continuous pedigree differentiation processes. This primordial cartilage is then cartilaginous in the cartilage growing plate, involved in bone elongation and temporary drive, or becomes permanent tissue, which is articular cartilage. The fate determinants and differentiation activities of chondrocytes are controlled by many extrinsic and intrinsic clues, which are achieved at the gene expression levels by transcription factors. Tissue repair capacity after injury is limited, often causing rheumatoid arthritis. MSC technology is a promising treatment strategy. SOX9 and RUNX2/3, as well as TWIST1, SOX5/6, and MEF2C/D, are the main transcription factors that regulate cartilage differentiation [67].

### 7.1. ZBED3-AS1

Ou et al. studied that the lncRNA expression profile changed during induced cartilage differentiation process in hBMSCs, which suggested that 2166 lncRNAs were upregulated and 1472 lncRNAs were downregulated; the expression of *ZBED3-AS1* (ZBED3 antisense RNA 1) and *CTA-941F9.9* was verified by Q-PCR. These results indicate that lncRNAs are involved in MSC cartilage differentiation [68] (Table 2).

### 7.2. DANCR

Zhang et al. found that *SOX4* in synovium-derived MSCs (SMSCs) can promote SMSC proliferation and differentiate into chondrocytes by upregulating lncRNA *DANCR*. *DANCR* (differentiation antagonizing non-protein-coding RNA) directly combines with *myc*, *Smad3*, and *STAT3* mRNA and regulates their stability. SMSC proliferation depends on *myc*. *DANCR* activates SMSC differentiation into chondrocytes by increasing Smad3 [69]. Zhang et al. found DANCR promoted chondrogenesis by downregulating the expression of miR-1305 which resulted in the decreased expression of Smad4 and activation of the TGF-*β* pathway in human synovium-derived stem cells (SMSCs) [70, 71] (Table 2).

### 7.3. HIT

Carlson et al. and Wang et al. found that lncRNA *HIT* was highly expressed in E11 mouse embryos, which is located in the nucleus and formed complexes with p100 and CBP. CHIRP-seq analysis revealed that the lncRNA-HIT-p100/CBP complex was associated with multiple sites in the mouse genome and exerted its role in cartilage differentiation. Silencing HIT with specific siRNA leads to decreased p100 activity and decreased H3K27ac, so lncRNA HIT plays an integral role in cartilage differentiation [72, 73] (Table 2).

## 8. lncRNAs Involved in Osteoclastogenesis

**Figure 3:**
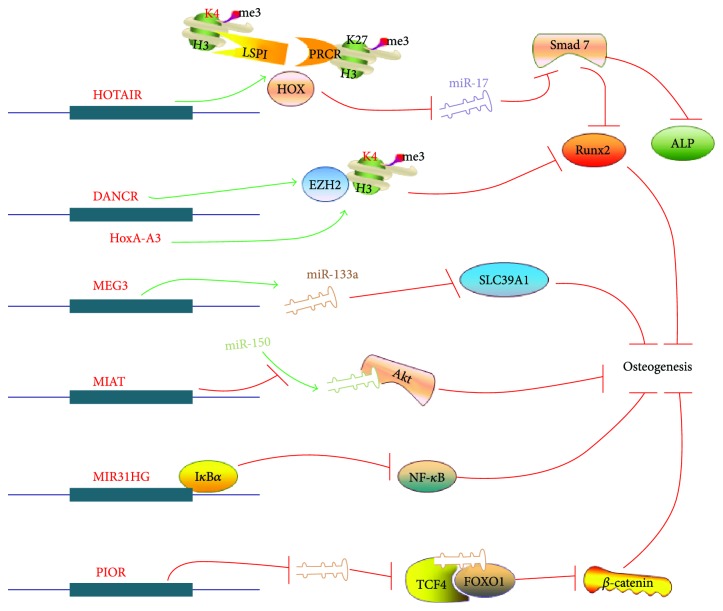
lncRNAs that contribute to lineage-specific phenotypes of MSCs.

Bone formation is a dynamic and continuous experience shaping, repair, and reconstruction process. Bone balance is mainly maintained which is dependent on osteoblasts involved in the formation of new bone and on osteoclasts involved in bone resorption. Osteoclasts are multinucleated cells derived from hematopoietic stem cells or monocyte/macrophage precursor cells. Osteoclast differentiation includes multiple stages. Dou et al. identified the transcriptional changes of RAW264.7 cells during osteoclast differentiation induced by RANKL (100 ng/mL) and M-CSF (50 ng/mL) for 24 h, 72 h, and 96 h. A series of changes were identified for circRNA, miRNA, lncRNA, and mRNA. The research group constructed 142 pairs of correlation between lncRNA and mRNA [74] and found that lncRNAs are also involved in the regulation of hematopoietic stem cell differentiation into osteoclasts. Osteoporosis is a common disease associated with reduced bone mineralization, which is mainly due to osteoblastic bone resorption exceeding bone formation function of osteoblasts. Tong et al. reported that lncRNA *DANCR* was involved in mononuclear cell formation in peripheral blood and was associated with human osteoporosis. *DANCR* promotes IL6 and TNF-*α* expression and increases bone resorption. These results suggest that lncRNAs are involved in bone resorption processes of osteoclasts [75] (Figure 3).

## 9. Concluding Remarks

In this manuscript, we summarized the long noncoding RNAs which play an important role in the osteogenic differentiation (*H19*, *MALAT1*, *MODR*, etc.), cartilage differentiation (*ZBED3-AS1*, *DANCR*, and *HIT*) from MSCs, and osteoclast differentiation (*DANCR)* from hematopoietic stem cells and mononuclear progenitor cells. The mechanism has been demonstrated. Compared with coding protein and small RNA, the knowledge of lncRNAs is only at an initial stage; functions and regulation mechanisms of which remain to be further elucidated. At present, we get to know about the functions and regulatory molecular mechanism through traditional techniques including in situ hybridization technology, overexpression technology, luciferase reporter gene system, and gene silencing technology by siRNA and Crisp/Cas9. At the same time, the development of some new technologies, such as CLIP (cross-linking immunoprecipitation) [76–78], RIP (RNA-binding protein immunoprecipitation) [79], RNA pulldown [80], CLASH (cross-linking, ligation, and sequencing of hybrids) [81], and ChIRP (chromatin isolation by RNA purifications) [82], has also provided a new platform for studying the networks involving proteins and RNA. With the development of more high-throughput screening technologies, such as microarray chip hybridization, combining the new generation of high-throughput sequencing technology with bioinformatics prediction tools, people will be able to find those with important regulatory functions more quickly and efficiently. The understanding, development, and application of the novel lncRNAs in the field of regeneration and repair will also present a new blueprint for a better and healthier life.

## Figures and Tables

**Table 1:** lncRNAs showing roles in osteogenic differentiation of stem cells.

Approved symbol	Aliases	Gene locus	In vitro observations	Mode of action	lncRNA-associated signaling	Stem cell category	Synonyms
H19	Imprinted maternally expressed transcript	11p15.5	Upregulated	Precursor of miR-675, sponging for miR-141 and miR-22	TGF-*β*1/Smad, Wnt/*β*-catenin	MSCs [49, 50]	ASM, ASM1, D11S813E, LINC00008
MALAT1	Metastasis associated lung adenocarcinoma transcript 1	11q13.1	Upregulated	Sponging for miR-204	Smad4	AVICs [52]	HCN, hepcarcin, LINC00047, MALAT-1, mascRNA, NEAT2
MODR	N/A	12q12	Upregulated	miR-140-5p, miR-454	RUNX2 suppression	hASCs, MSMSCs [53]	ENST00000537192.1
POIR	N/A	6q21-q22.1	Upregulated	Sponging for miR-182	Wnt/*β*-catenin, NF-*κ*B	hBMSCs [65]	ENST00000421891
HOTAIR	HOX transcript antisense RNA	12q13.13	Downregulated	Sponging for miR-204 and miR-17-5p, histone modification	H3K27me3 increase and H3K4me3 decrease	MMPs [32, 54]	HOXC-AS4, HOXC11-AS1, NCRNA00072
DANCR	Differentiation antagonizing non-protein-coding RNA	4q12	Downregulated	Sponging for mir-4449	H3K27me3 increases	PHKC, hFOB1.19 [55, 56]	Adipogenesis upregulated transcript 2, AGU2, ANCR, and lncRNA ANCR
MEG3	Maternally expressed 3	14q32.2	Upregulated Downregulated	Sponging for mir-140-5p and miR-133a-3p	BMP4	hBMSCs, ASCs [57–59]	GTL2, LINC00023, NCRNA00023, nco-lncRNA-83
HoxA-AS3	HOXA cluster antisense RNA 3	7p15.2	Downregulated	Interacting with EZH2	H3K27me3 increase	HBMSCs [60, 61]	HOXA6as
MIAT	Myocardial infarction-associatedtranscript	22q12.1	Downregulated	Sponging for miR-150-5p	AKT	hASCs [62–64]	FLJ25967, Gomafu, LINC00066, lncRNA MIAT
MIR31HG	LOC554202	9p21.3	Downregulated	Interacting with I*κ*B*α*	NF-*κ*B activation	hASCs [66]	ENSG00000171889, RP11-354P17.9, MIR31HG, LOC554202, hsa-lnc-31

MSMSCs: maxillary sinus membrane stem cells; PMSCs: periodontal mesenchymal stem cells of periodontitis patients; PHKC: primary human keratinocytes; hFOB1.19: human fetal osteoblastic cell line; hBMSCs: human bone marrow-derived mesenchymal stem cells; hASCs: human adipose-derived stem cells; ACIC: aortic valve interstitial cells.

**Table 2:** lncRNAs showing roles in chondrocyte differentiation of stem cells.

Approved symbol	Aliases	Gene locus	In vitro observations	Mode of action	lncRNA-associated signaling	Stem cell category
DANCR	Differentiation antagonizing nonprotein	4q12	Upregulated	Sponging for miR-1305-Smad4	Sox4 binds to the promoter	SMSCs, hSDSC [70, 71]
ZBED3-AS1	5q13.3		Upregulated	Directly increases zbed3 expression	Activates Wnt/*β*-catenin signaling	hSFMSC [69]
lncRNA HIT	—	Chr6	Upregulated	Forms a complex with p100 and CBP	Increases H3K27ac or p100 activity	Early limb development in mice [72, 73]
